# An in-depth analysis of glycosylated haemoglobin level, body mass index and left ventricular diastolic dysfunction in patients with type 2 diabetes

**DOI:** 10.1186/s12902-019-0419-7

**Published:** 2019-08-27

**Authors:** Xin Zuo, Xueting Liu, Runtian Chen, Huiting Ou, Jiabao Lai, Youming Zhang, Dewen Yan

**Affiliations:** 1 Department of Endocrinology, Xiangya-Shenzhen Endocrinology and Metabolism Center, the First Affiliated Hospital of Shenzhen University, Shenzhen, People’s Republic of China; 2 Genomic and Environmental Medicine Section, National Heart and Lung Institute, Imperial College London, Dovehouse Street, London, SW3 6LY UK

**Keywords:** Type 2 diabetes, Left ventricular diastolic dysfunction, Glycosylated haemoglobin, Body mass index

## Abstract

**Background:**

Glycosylated hemoglobin (HbA1c) has a detrimental impact on the myocardium with left ventricular (LV) diastolic dysfunction. Obesity is a risk factor of type 2 diabetes. To understand the relationships between HbA1c, body mass index (BMI) and LV diastolic dysfunction, we performed this interaction analysis in patients with type 2 diabetes.

**Methods:**

Total 925 type 2 diabetes patients were selected from the patients who were diagnosed and treated at the First Affiliated Hospital of Shenzhen University. Patients’ BMI levels were defined as normal (BMI < 24 kg/m^2^) and overweight /obese (BMI ≥ 24 kg/m^2^). Patients’ HbA1c levels were grouped as HbA1c ≥ 9%、7% ≤ HbA1c < 9% and HbA1c < 7%. Logistic regression, stratified, interaction analysis, multivariate Cox regression and curve fitting analysis were performed to investigate the correlations and interactions between HbA1c and BMI with LV diastolic dysfunction.

**Results:**

The BMI levels were significantly associated with LV diastolic dysfunction in the patients with type 2 diabetes [adjusted model: 1.12 (1.05, 1.20)*, P* = 0.001]. While HbA1c levels had association with LV diastolic dysfunction only in normal BMI group patients [adjusted model: 1.14 (1.01, 1.30), *P* = 0.0394] and curve correlation was observed. There was a significant interaction between BMI and HbA1c to affect LV diastolic dysfunction (*P* = 0.0335). Cox regression model analysis showed that the risk of LV diastolic dysfunction was a U type correlation with HbA1c levels in the normal weight group and the turning point was HbA1c at 10%. HbA1c level was not found to have a significant association with LV diastolic dysfunction in overweight/obese group.

**Conclusions:**

In patients with type 2 diabetes, correlation between LV diastolic dysfunction and HbA1c was interactively affected by BMI. Glycemic control is beneficial to the heart function in normal body weight patients. For overweight/obese patients, the risk of LV diastolic dysfunction was not determined by the HbA1c level, indicating it may be affected by other confounding factors.

## Background

Diabetes is one of the most important health problems around the world in the twenty-first century [1]. The disease can cause damages in microvessels of multiple organs such as heart and kidney. Cardiovascular complications are the leading causes of death in patients with diabetes [2]. Diabetic cardiomyopathy (DCM) is generally considered to be the manifestation of microvascular disease in the heart [3]. The left ventricular (LV) diastolic dysfunction is the earliest functional change in DCM, and eventually progresses to heart failure with normal cardiac ejection fraction [4]. Hyperglycaemia in patients with diabetes can have much detrimental impacts on the myocardium form lipotoxicity, mitochondrial dysfunction, abnormal substrate metabolism and impaired calcium handling [5–7]. Glycosylated hemoglobin (HbA1c) can reflect the average level of glycemic control. The glycosylation occurs by the non-enzymatic reaction between glucose and N-terminal end of the β-chain of hemoglobin [8]. An average of 1% reduction in mean HbA1c levels could reduce 37% microvascular complications [9]. Glycosylated hemoglobin plays an important role in the assessment of the occurrence, development and prognosis of diabetic cardiovascular complications. The high incidence of diastolic dysfunction was found in asymptomatic diabetes correlating with HbA1c levels in Indian patients [10].

Overweight and obesity have long been regarded as health risks associated with the type 2 diabetes [11]. Overweight and obesity itself not only can lead to left ventricular dilatation, hypertrophy, impaired relaxation and diastolic dysfunction; but also can cause aggravated insulin resistance, increase the difficulty of blood glucose control and increase the risk of cardiovascular disease. BMI can reflect the level of weight control. Until now, there was no report to focus on interaction analysis of body mass index (BMI), HbA1c levels and LV diastolic dysfunction in patients with type2 diabetes in China; therefore we performed this investigation in a cohort of patients with type 2 diabetes in a region of South China.

## Methods

### Population and data

Total of 1440 patients with type 2 diabetes were diagnosed at the Endocrinology Department from January 2017 to October 2018 in the First Affiliated Hospital of Shenzhen University of Guangdong Province, South China. All information of the patients was recorded in a bioinformatics database called β-CVK (β-function; C-cardiac; V-vascular; K-kidney). The database was established for evaluation of the functions of major organs such as pancreas islet, heart and kidney in patients with diabetes. Total 925 cases were selected in the current investigation with our inclusion criteria including: 1) Patients were diagnosed as type 2 diabetes according to America Diabetes Association definition [12]; 2) Patients had complete data records such as age, height, weight, waist circumference, laboratory examinations and underwent a resting standard echocardiographic examination. Patients were excluded from the investigation if they met any of the following conditions: 1) Patients’ ejection fraction (EF) was < 50%; 2) Patients had previous or current history of coronary artery diseases, congenital heart diseases, heart surgery and other heart diseases; 3) BMI < 18.5 kg/ m^2^. 4) Patients had other serious diseases such as hematological tumors and uremia; 5) Patients had acute metabolic disorders including diabetic ketoacidosis and hyperosmolar coma. 6) Pregnant patients. 7) Patients with renal insufficiency (eGFR < 60 mL/min/1.73m^2^).

The study was approved by the Ethics Committee of the First Affiliated Hospital of Shenzhen University, Shenzhen, China and was performed in accordance with the approved guidelines and regulations. Informed written consent was obtained from the parents of all subjects.

The selected patients were recorded with general information. BMI was divided into two different groups according to the Chinese expert consensus standards as normal weight group and overweight/obese group, using the thresholds of BMI < 24 kg/m^2^ and BMI ≥24 kg/m^2^, the standard was modified form the criteria applied by American College of Cardiology [13]. HbA1c level was detected by applying high performance liquid chromatography with the detection instrument I of BIO-RAB-D10 glycated hemoglobin detector; triglyceride (TG), total cholesterol (Cho), low density lipoprotein cholesterol (LDL-C), high Density lipoprotein cholesterol (HDL-C) and creatinine (Cr) were measured by oxidase methods; homocysteine (HCY) was enzymatically cycled, all the measurements were carried out in strict accordance with the instructions of the kit; estimated glomerular filtration rate (eGFR) was simplified MDRD formula calculation: eGFR [mL / (min • 1.73m^2^)] = 186 × (Cr / 88.4) - 1.154 × age - 0.203 × 0.742. Cardiac diastolic function parameters were measured by echocardiography (PHILIPS EPIQ-7C), and LV diastolic peak filling flow rate (Peak E), LV end-diastolic peak filling flow rate (Peak A) were recorded. Peak E/A ratio less than 1 was considered as LV diastolic dysfunction together with other measurements by agreements with two experienced ultrasonic consultants [14].

### Data analysis

Statistical analysis was performed by using SPSS 21.0 statistical software (IBM® SPSS Statistics, New York, USA) and EmpowerStats (http://www.empowerstats.com, X&Y Solutions, Inc., Boston, MA). Data were presented as mean ± standard deviation (SD) or median (interquartile) for continuous variables, and as frequency or percentage for categorical variables. The t tests and chi-square tests were used to determine any statistical difference between the means and proportions of the two groups. Single-factor analysis of variance was used for comparison between groups. Univariate and multiple logistic regression models were used to evaluate the associations between HbA1c levels, the BMI, and LV diastolic dysfunction. Both non-adjusted and multivariate adjusted models (variables adjusted for age, sex, smoking status, drinking status, systolic blood pressure, diastolic blood pressure and course of disease, glomerular filtration rate, triglyceride and uric acid) were applied. Interaction and stratified analysis were conducted according to HbA1c levels (HbA1c ≥ 9%; 7% ≤ HbA1c < 9%, HbA1c < 7%) and BMI status (BMI < 24 kg/m^2^; BMI ≥ 24 kg/m^2^). Curve-fitting analysis was performed to further clarify the action trend of HbA1c levels and LV diastolic dysfunction in different BMI groups. A priori interactions between BMI and HbA1c using the multivariable Cox regression model was analysed to further assess whether the relationship between HbA1c and LV diastolic dysfunction varied by BMI status. *P* value < 0.05 was considered as statistically significant.

## Results

### Subjects and clinical characteristics

Among 925 patients with type 2 diabetes in this study, there were 391 cases with normal body weight (BMI < 24 kg/m^2^), 534 cases with overweight/ obese (BMI ≥ 24 kg/m^2^). There were no significant differences in age, total cholesterol, high-density lipoprotein, low-density lipoprotein, eGFR, HbA1c levels between two groups (*P* > 0.05). Overweight/obese group had more smokers and alcohol drinkers than normal weight group (44.57% vs 35.55, 33.52% vs 21.23% respectively). Overweight/obese patients had higher blood pressure, higher serum triglycerides, higher uric acid and 24-h urinary but lower high-density lipoprotein levels (*P* <  0.05). The proportion of LV diastolic dysfunction was higher in overweight/obese group (68.29% vs 75.09%, *P* = 0.0022). The ejection fraction (EF value) was not statistically significant between the two groups (Table 1).

**Table 1:** Clinical characteristics between normal weight group and overweight/obesity group in patients with type 2 diabetes

Characteristic	Normal weight *N*(%)/Mean ± SD	Overweight/Obese *N*(%)/Mean ± SD	*P* value
Cases	391	534	
Sex (male)	247 (63.17%)	405 (75.84%)	< 0.001a
Age (years)	57.09 ± 11.86	57.61 ± 11.67	0.059b
Course of disease (years)	10.81 ± 7.13	9.85 ± 7.09	0.035b
Smoking	139 (35.55%)	238 (44.57%)	0.022a
Alcohol drinking	83 (21.23%)	179 (33.52%)	< 0.001a
BMI (kg/m^2^)	22.16 ± 1.32	26.79 ± 2.15	< 0.001b
Systolic pressure (mmHg)	127.12 ± 20.16	131.71 ± 16.91	< 0.001b
Diastolic pressure (mmHg)	76.03 ± 10.70	79.48 ± 10.10	< 0.001b
Triglyceride (mmol/L)	1.76 ± 0.86	2.07 ± 1.12	< 0.001b
Total cholesterol (mmol/L)	4.34 ± 1.12	4.46 ± 1.64	0.232b
High-density lipoprotein (mmol/L)	0.96 ± 0.24	0.98 ± 0.21	0.217b
Low-density lipoprotein (mmol/L)	2.67 ± 0.87	2.66 ± 0.90	0.823b
Glycosylated hemoglobin(%)	8.86 ± 2.10	8.68 ± 2.02	0.185b
Uric acid (umol/L)	345.80 ± 83.39	389.45 ± 97.26	< 0.001b
eGFR (mL/min/1.73m^2^)	112.11 ± 37.10	111.79 ± 54.13	0.083b
24-h urinary albumin (mg/24 h)	97.78 ± 346.39	161.16 ± 537.57	< 0.001b
Left ventricular diastolic dysfuction)	267 (68.29%)	401 (75.09%)	0.022a
Ejection fraction(%)	66.71 ± 4.78	66.38 ± 4.78	0.308b

a: *P* values of categorical variables were obtained by the chi-square test

b: *P* values of continuous variables were obtained by student t test

We then compared the baseline characteristics of normal LV diastolic function group with LV diastolic dysfunction group. There were no significant differences in HbA1c level, triglyceride, total cholesterol, high-density lipoprotein, low-density lipoprotein, ejection fraction, drinking history, diastolic blood pressure between two groups (*P* > 0.05). The age of diastolic dysfunction group was older (mean age was 47.08 ± 10.44 years in the normal group and 59.17 ± 10.77 years in the dysfunction group; *P* <  0.001), and the course of disease was longer (7.68 ± 6.24 years in the normal group and 11.34 ± 7.07 years in the dysfunction group, *P* <  0.001). Systolic blood pressure in LV diastolic dysfunction group was higher in the dysfunction group (mean systolic blood pressure was 124.81 ± 15.84 mmHg in the normal group and 131.42 ± 19.09 mmHg in the dysfunction group; *P* <  0.001), the systolic blood pressure was controlled within the normal range (Table 2).

**Table 2:** Clinical characteristics between normal LV diastolic function and left ventricular diastolic dysfunction in patients with type 2 diabetes

Characteristic	Normal left ventricular diastolic function *N*(%)/Mean ± SD	Left ventricular diastolic dysfunction *N*(%)/Mean ± SD	*P* value
Cases	257 (27.78%)	668 (72.22%)	
Sex (male)	190 (71.43%)	391 (62.26%)	0.009a
Age (years)	47.08 ± 10.44	59.17 ± 10.77	< 0.001b
Course of disease (years)	7.68 ± 6.24	11.34 ± 7.07	< 0.001b
Smoking	117 (43.98%)	227 (36.15%)	0.028a
Alcohol drinking	77 (28.95%)	154 (24.52%)	0.167a
Systolic pressure (mmHg)	124.81 ± 15.84	131.42 ± 19.09	< 0.001b
Diastolic pressure (mmHg)	78.34 ± 9.87	77.28 ± 10.61	0.163b
HbA1c(%)	8.91 ± 2.18	8.67 ± 1.95	0.100b
Triglyceride (mmol/L)	2.08 ± 1.13	2.06 ± 1.14	0.813b
High-density lipoprotein (mmol/L)	0.97 ± 0.23	0.99 ± 0.24	0.551b
Low-density lipoprotein (mmol/L)	2.67 ± 0.85	2.55 ± 0.93	0.057b
BMI (kg/m2)	24.23 ± 3.03	25.12 ± 3.02	< 0.001b
Ejection fraction(%)	66.34 ± 4.55	66.82 ± 4.80	0.182b

a: *P* values of categorical variables were obtained by the chi-square test

b: *P* values of continuous variables were obtained by student t test

### Regression analysis of BMI levels, HbA1c levels and LV diastolic dysfunction in patients with type 2 diabetes

We applied logistic regression to analyze the BMI levels and LV diastolic dysfunction. We applied two models for the investigation: Non-adjusted model and adjusted model (with gender, age, smoking status, drinking status, systolic blood pressure, diastolic blood pressure and course of disease, glomerular filtration rate, triglyceride and uric acid). In general, we found BMI levels from patients with type 2 diabetes had associations with LV diastolic dysfunction in two models [non-adjusted model: 1.07 (1.02, 1.13), *P* = 0.0059, adjusted model: 1.12 (1.05, 1.20), *P* = 0.0010)]. Comparing with the normal weight group, the risk ratio (OR value) was 1.40 (1.05, 1.87), *P* = 0.087 in overweight/obese group with non-adjusted model and was 1.71 (1.17, 2.50), *P* = 0.0056 with adjusted model.

We then applied logistic regression to analyze the HbA1c levels and LV diastolic dysfunction with type 2 diabetes. There were not any association both in non-adjusted model [0.95 (0.89, 1.02), *P* = 0.1589] and adjusted model [0.99 (0.91, 1.08), *P* = 0.8601] (Table 3).

**Table 3:** Logistic regression analysis of BMI, HbA1c and LV diastolic dysfunction in patients with type 2 diabetes

Exposure	Non-adjusted model OR(95% CI) *P* value	Adjusted model OR(95% CI) *P* value
BMI (kg/m^2^) (Analysis by continuous variables)	1.07 (1.02, 1.13) 0.0059	1.12 (1.05, 1.20) 0.0010
BMI (kg/m^2^) (Analysis by categorical variables)		
BMI < 24(kg/m^2^)	1.0(ref.)	1.0(ref.)
BMI ≥ 24(kg/m^2^)	1.40 (1.05, 1.87) 0.0227	1.71 (1.17, 2.50) 0.0056
HbA1c(%) (Analysis by continuous variables)	0.95 (0.89, 1.02) 0.1589	0.99 (0.91, 1.08) 0.8601
HbA1c (Analysis by categorical variables)		
HbA1c < 7%	1.0(ref.)	1.0(ref.)
7% ≤ HbA1c < 9%	1.07 (0.72, 1.60) 0.7210	0 .90 (0.56, 1.47) 0.6855
HbA1c ≥ 9%	0.82 (0.55, 1.20) 0.3043	1.03 (0.63, 1.67) 0.9120

Adjusted model: adjusted for age, sex, smoking status, drinking status, systolic blood pressure, diastolic blood pressure and course of disease, glomerular filtration rate,triglyceride and uric acid

*OR* Odds ratio, *CI* Confidence interval

### Stratified and curve fitting analysis of HbA1c and LV diastolic dysfunction in patients with type 2 diabetes

To further explore whether the relationship between HbA1c and LV diastolic dysfunction in type 2 diabetes is affected by BMI, stratified analysis was performed (Table 4). We found that HbA1c was significantly associated with LV diastolic dysfunction in the normal weight group (OR = 1.14 (1.01, 1.30), *P* = 0.0394), whereas not in overweight/obese group (OR = 0.92 (0.82, 1.03), *P* = 0.1315), suggesting that the association between HbA1c and left ventricular diastolic dysfunction is affected by BMI levels.

**Table 4:** Stratified analysis of HbA1c and LV diastolic dysfunction in patients with type 2 diabetes

Exposure	BMI<24 kg/m^2^ OR (95%CI) *P* value	BMI ≥24 kg/m^2^ OR(95% CI) *P* value
HbA1c(%)	1.14 (1.01, 1.30) 0.0394	0.92 (0.82, 1.03) 0.1315
HbA1c		
HbA1c < 7%	1.0(Ref.)	1.0(Ref.)
7% ≤ HbA1c < 9%	0.71 (0.35, 1.47) 0.3632	0.97 (0.52, 1.82) 0.9356
HbA1c ≥ 9%	1.86 (0.90, 3.82) 0.0928	0.71 (0.39, 1.31) 0.2765

*OR* Odds ratio, *CI* Confidence interval, *Ref* Reference

**Fig. 1:**
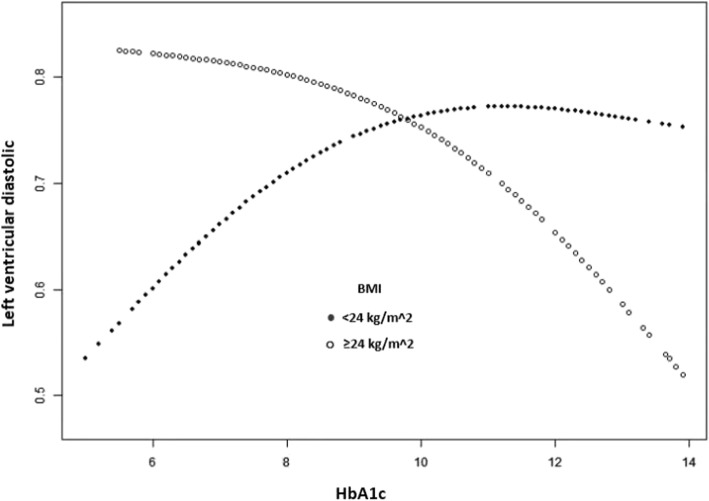
Relationship between HbA1c and LV diastolic dysfunction in patients with type 2 diabetes at different BMI states. Curve fitting analysis was performed in the two BMI groups for LV diastolic dysfunction. y -axes: Left diastolic function; x-axes: HbA1C levels

We further performed curve fitting analysis for the two BMI groups for LV diastolic dysfunction; the results were shown in Fig. 1. The relationships between HbA1c levels and LV diastolic dysfunction were different at different BMI state: in normal body weight group, HbA1C levels and LV diastolic function had a curve relationship, before the curve inflection point (about 10%), the risk of LV diastolic dysfunction increased when HbA1C level increased, after the point, the HbA1c level did not affect LV diastolic dysfunction any more. In overweight/obesity group, the baseline risk of LV diastolic dysfunction in these patients is already high even if the level of HbA1c was low, and the HbA1c level was no longer a key factor to contribute the risk.

### Interaction analysis of BMI, HbA1c levels on LV diastolic dysfunction in patients with type 2 diabetes

**Fig. 2:**
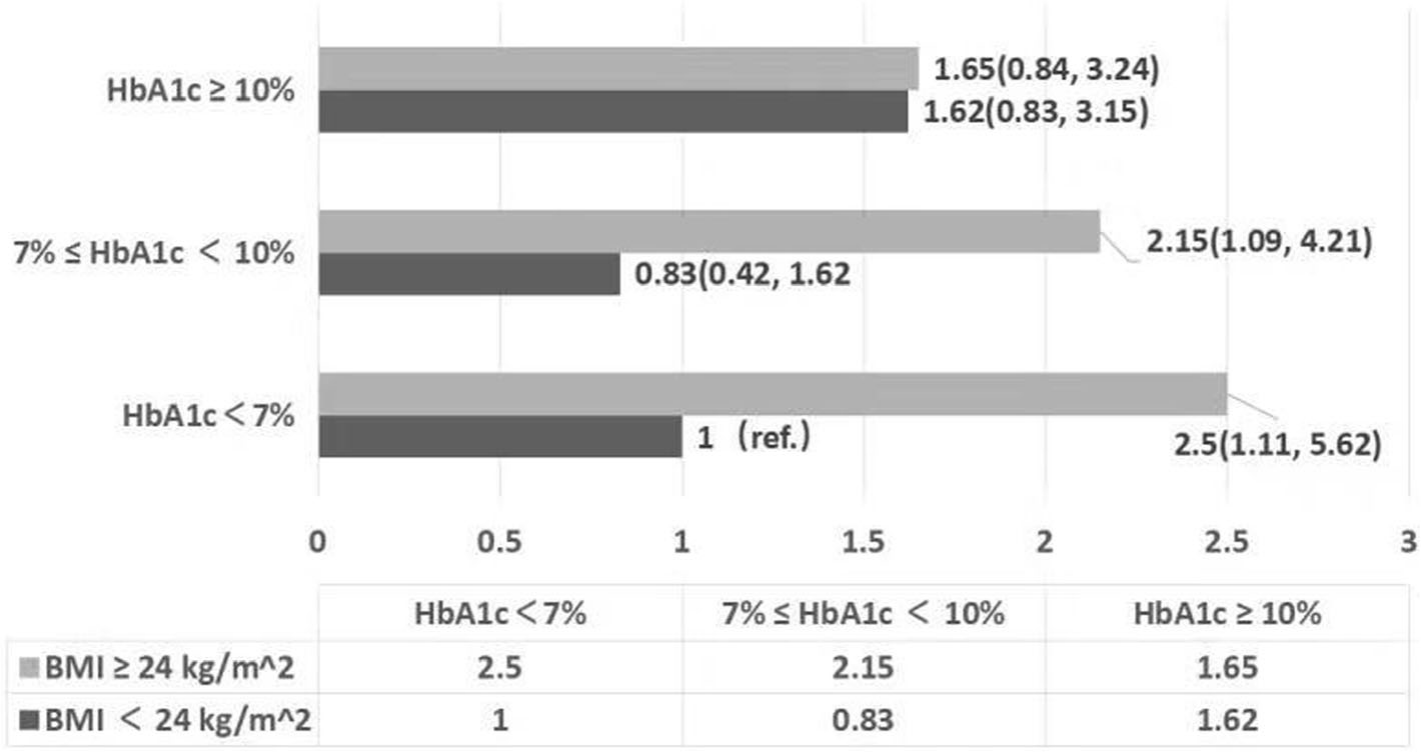
BMI and HbA1c subgroup and LV diastolic dysfunction in patients with type 2 diabetes. Multivariable Cox regression model to assess the role of each subgroup model combination in left ventricular diastolic dysfunction. A subgroup model combining normal body weight (BMI < 24 kg/m^2^) with HbA1c < 7%was used as a reference

Interaction analysis revealed that there was a significant interaction between BMI and HbA1c level to affect LV diastolic function (*P* = 0.0371). The results were shown in Table 5. We then applied multivariable Cox regression to assess the role of each subgroup model combination in LV diastolic dysfunction. We took subgroup model combining normal body weight (BMI < 24 kg/m^2^) with HbA1c < 7% as the reference. The results were shown in Fig. 2. In the normal weight group, the risk of LV diastolic dysfunction presented an U-shaped curve as the increase of HbA1c level the ORs in HbA1c ≥ 9%,7% ≤ HbA1c < 9% and HbA1c < 7% group were 1.62, 0.82, 1 respectively, which was consistent with previous study [8]. In the overweight/obese group, no positive correlation was found. The combination of overweight/obese group and HbA1c < 7% had the greatest risk of LV diastolic dysfunction [OR = 2.50 (1.11, 5.62), *P* = 0.0271]. It may suggest that HbA1c < 7% is no longer the optimal control threshold in the management of cardiovascular complications in overweight/obese patients.

**Table 5:** Interaction Analysis of the BMI and HbA1c in LV diastolic dysfunction in patient with type 2 diabetes

Exposure	HbA1c level	OR (95% CI) *P* value
BMI < 24 (kg/m^2^)	HbA1c < 7%	Ref.
BMI ≥ 24 (kg/m^2^)	HbA1c < 7%	2.50 (1.11, 5.62) 0.0176
BMI < 24 (kg/m^2^)	7% ≤ HbA1c < 9%	0.83 (0.42, 1.62) 0.5782
BMI ≥ 24 (kg/m^2^)	7% ≤ HbA1c< 9%	2.15 (1.09, 4.21) 0.0262
BMI < 24 (kg/m^2^)	HbA1c ≥ 9%	1.62 (0.83, 3.15) 0.158
BMI ≥ 24 (kg/m^2^)	HbA1c ≥ 9%	1.65 (0.84, 3.24) 0.1443
*P* interaction		0.0371

*OR* Odds ratio, *CI* Confidence interval, *Ref* Reference. *P* interaction: *P* value was calculated for comparison between subgroups

## Discussion

The incidence of type 2 diabetes has rapidly increased over recent decades and become one of leading public health problems in China. One third of patients with heart failure have diabetes. This is partly because all the major risk factors for heart failure can present in patients with type 2 diabetes such as obesity, hypertension, advanced age, sleep apnoea, dyslipidaemia, anemia, chronic kidney disease, and coronary heart disease [15]. Hyperglycaemia is a risk factor for heart failure in persons with type 2 diabetes [16]. Excess body weight, is also a major risk factor for cardiovascular disease [17].

Understanding the relationships between BMI levels, HbA1c levels and LV diastolic dysfunction in the patients with type 2 diabetes will help clinicians to have appropriate management plans for the patients and reduce heart failure rates in patients. In this investigation, we had an in-depth analysis of the relationships among BMI, HbA1c and LV diastolic dysfunction of 925 type 2 diabetes patients in South China. We had found that the BMI was an independent risk factor to LV diastolic dysfunction, confirming previous reports from South Korea [18], Australia and the Netherlands [19], and USA [20]. Chinese population was transformed from one of the leanest populations to have more than 200 million overweight or obese in its population [21]. Our results of the independent role of BMI to LV diastolic dysfunction in type 2 diabetes indicates the importance of education of health diets, exercises to prevent the most common heart diseases in China.

Hyperglycaemia has detrimental effects on the myocardium [5]. It up-regulates renin-angiotensin-aldosterone system, increases oxidative stress [22], promotes accumulation of glycation end-products and causes interstitial fibrosis in heart muscle [23]. Hyperglycaemia has strong associations with myocardial lipotoxicity, mitochondrial dysfunction, abnormal substrate metabolism and impaired calcium handling [6]. The HbA1c level is now recommended as a standard for testing and monitoring diabetes [24]. Previous investigations from DCCT, ADVANCE and UKPDS showed that the risk of microangiopathy in patients with type 2 diabetes increased with the increase of HbA1c levels. The microangiopathy in these studies were mostly diabetic nephropathy, diabetic retinopathy and neuropathy [25–27]. The relationship between HbA1c and LV diastolic dysfunction has been reported as contradiction. Giorda et al. found that HbA1c was significantly correlated with LV diastolic function in patients with type 2 diabetes [28], Clarke et al. also found that LV diastolic dysfunction is inversely linked to glycemic control in T2DM patients [29]. Noh et al. did not found that HbA1c increased risk of LV diastolic dysfunction in type 2 diabetes [30]. The different results may be related to the different blood glucose control status of the included populations. In our present study, we did not found significant correlation between HbA1c level and LV diastolic dysfunction in patients with type 2 diabetes. Our results suggest that HbA1c effects on LV diastolic dysfunction may be affected by some other confounding factors. Obesity is becoming increasingly prominent, not only affecting cardiovascular health, but also leading to glucose and lipid metabolism disorder. Stratified analysis in our investigation found that HbA1c was statistically correlated with LV diastolic dysfunction only in the normal weight group, suggesting that obesity is an important confounding factor in the relationship between HbA1c and LV diastolic dysfunction. In the normal weight group, we found that the relationship between HbA1c and LV diastolic dysfunction was curvilinear correlation, and had a saturation threshold (10%). In a Scotland cohort study of 8683 patients with type 2 diabetes, both high and low HbA1c levels have been found to predict heart failure development forming a U-shaped relationship [8]. It may suggest that under normal weight, proper glycemic control is beneficial to the improvement of heart function. When HbA1c is higher than a certain range (10%) or in overweight/obesity, glycemic control may no longer be the most important factor affecting LV diastolic dysfunction. More attention should be paid to the some other effects such as lipotoxicity on cardiac function. In a Western Indian study, the dyslipidemic obese subjects had significant linear association with HbA1c in type 2 diabetes subjects [31]. A rise in obesity and dyslipidemia with increasing urbanization have led to various lifestyle related disorders like type 2 diabetes, cardiovascular disease (CVD) and metabolic syndrome. Among dyslipidemic subjects, increased LDL-C, TG and hypo-HDL are established markers for CAD risk. The other reason is that HbA1c-lowering is greater in populations with poor glycaemic control than in well controlled populations with the same degree of weight loss [32]. The genetic background for type 2 diabetes may be different in South China and may be relevance to the complex phenotypes of HbA1c phenotypes of patients with type 2 diabetes. In overweight/obese group, analysis found the obesity paradox: increased HbA1c unexpectedly reduced the risk of diastolic dysfunction, which was similar to some previous obesity paradox studies [33–35] . There may be some other unmeasured confounders not yet be excluded, or because obesity itself may be a risk factor for disease events, and stratification bias can amplify the effects of confounding factors. The specific causes still need to be further investigated.

In this study, it was found for the first time that BMI and HbA1C had significant interactions in influencing the left ventricular diastolic dysfunction, suggesting that the interaction of multiple factors should be considered in the management of cardiac complications in type 2 diabetes mellitus.

There were some limitations in this study. The current investigation applied the evaluation index using the E/A ratio in the echocardiogram, it did not investigate the state of the heart load; the HbA1c levels which represented glycemic control in this study, only corresponded to changes in blood glucose levels for nearly 3 months, while diabetes cardiomyopathy and obesity are metabolic diseases and the pathological processes that change gradually over a long period of time. Single HbA1c may not reflect the historical impact of hyperglycemia on diastolic dysfunction. The effects of some medications and other confounders such as blood pressure and blood lipids were not fully considered, these confounders are needed to further research in detail.

The current investigation provides clinical implication about the importance of weight control in patients with type 2 diabetes as higher BMI was clearly a risk of LV diastolic dysfunction. It also indicates monitoring both HbA1c levels and BMI levels could assist us for correctly predicting early heart function changes for the patients. The results can help clinicians for appropriate management of patients with type 2 diabetes.

## Conclusions

In patients with type 2 diabetes, the correlation between LV diastolic dysfunction and HbA1c was interactively affected by BMI levels. Under normal body weight, proper glycemic control is beneficial to protect the heart function when HbA1c is less than 10%, Under overweight/obese condition, the risk of LV diastolic dysfunction in these patients may not related to the HbA1c level, indicating weight loss could bring cardiovascular benefits in the patients with type2 diabetes.

## Availability of data and materials

The experimental datasets in the analysis described in this study are available from Professor Dewen Yan or Dr. Xin Zuo.

## Data Availability

The original data were recorded in our cohort database and are available if required.

## Abbreviations

BMI: Body mass index
CVD: Cardiovascular disease
DCM: Diabetic cardiomyopathy
EF: Ejection fraction
eGFR: Estimated glomerular filtration rate
HbA1c: Hemoglobin A1c or glycosylated hemoglobin
HDL-C: High Density lipoprotein cholesterol
LDL-C: Low density lipoprotein cholesterol
LV: Left ventricular
TG: Triglyceride
